# Retraction Note: Dysregulated JAK2 expression by TrkC promotes metastasis potential, and EMT program of metastatic breast cancer

**DOI:** 10.1038/s41598-025-18790-5

**Published:** 2025-09-29

**Authors:** Min Soo Kim, Joon Jeong, Jeongbeob Seo, Hae-Suk Kim, Seong-Jin Kim, Wook Jin

**Affiliations:** 1 Laboratory of Molecular Disease and Cell Regulation, Department of Biochemistry, School of Medicine, GachonUniversity, Incheon, 406-840 Korea; 2 Department of Surgery, Gangnam Severance Hospital, Yonsei University Medical College, 712 Eonjuro, Gangnam-Gu, Seoul, 135-720 Korea; 3 Medicinal Chemistry, CMG Pharma, 335, CHA Bio Complex, Pangyo-ro, Bundang-gu, Seongnam-si, Gyeonggi-do, 13488 Korea; 4 TheragenEtex Bio Institute, TheragenEtex Co., Suwon, Gyeonggi-do 16229 Republic of Korea; 5 Nano-Bio Medicine Research Center, Advanced Institutes of Convergence Technology, and Department of Transdisciplinary Studies, Graduate School of Convergence Science and Technology, Seoul National University, Suwon, Kyunggi-do 16229 Republic of Korea; 6 Gachon Medical Research Institute, Gil Medical Center, Incheon, 405-760 Korea

Retraction of: *Scientific Reports* 10.1038/srep33899, published online 22 September 2016

The Editors have retracted this Article. After publication, concerns were raised regarding highly similar data between this Article and the authors’ earlier study^1^, specifically:

- Fig. 4d JAK2 and P-STAT3 blots appear highly similar to Fig. 4d STAT3 and P-STAT3, respectively, in^1^;
- Fig. 6d N-Cadherin and DAPI + merge MDCK images appear highly similar to those in Fig. 5d of^1^;
- Fig. 6e Goosecoid blot lane 1 appears highly similar to Fig. 5e Goosecoid lane 2 in^1^;
- Fig. 6e Gapdh blot appears highly similar to Fig. 5e Gapdh in^1^.

Additionally, Fig. 2c β-actin appears highly similar to Fig. 3g left β-actin lanes 2–4.

The Authors have provided partial raw data upon request, but have been unable to share the original blot scans for the affected figures.

The Editors therefore no longer have confidence in the presented data.

Min Soo Kim, Joon Jeong, Seong-Jin Kim and Wook Jin agree with this retraction. Hae-Suk Kim has not responded to any correspondence from the Editors about this retraction. The Editors have not been able to obtain a current email address for Jeongbeob Seo.

## References

[1] Kim M., Lee W., Jeong J., Kim S. & Jin W. Induction of metastatic potential by TrkB via activation of IL6/JAK2/STAT3 and PI3K/AKT signaling in breast cancer. *Oncotarget*. **6**, 40158–40171. 10.18632/oncotarget.5522 (2015). 10.18632/oncotarget.5522 PMC4741886 26515594

